# SET_ER/PR_: a robust 18-gene predictor for sensitivity to endocrine therapy for metastatic breast cancer

**DOI:** 10.1038/s41523-019-0111-0

**Published:** 2019-05-30

**Authors:** Bruno V. Sinn, Chunxiao Fu, Rosanna Lau, Jennifer Litton, Tsung-Heng Tsai, Rashmi Murthy, Alda Tam, Eleni Andreopoulou, Yun Gong, Ravi Murthy, Rebekah Gould, Ya Zhang, Tari A. King, Agnes Viale, Victor Andrade, Dilip Giri, Roberto Salgado, Ioanna Laios, Christos Sotiriou, Esmeralda C. Marginean, Danielle N. Kwiatkowski, Rachel M. Layman, Daniel Booser, Christos Hatzis, V. Vicente Valero, W. Fraser Symmans

**Affiliations:** 1 Department of Pathology and Translational Molecular Pathology, The University of Texas MD Anderson Cancer Center, Houston, TX USA; 2 Department of Pathology, Charité-Universitätsmedizin Berlin, corporate member of Freie Universität Berlin, Humboldt-Universität zu Berlin, and Berlin Institut of Health, Berlin, Germany; 3 Department of Breast Medical Oncology, The University of Texas MD Anderson Cancer Center, Houston, TX USA; 4 Department of Interventional Radiology, The University of Texas MD Anderson Cancer Center, Houston, TX USA; 5 Department of Medicine, Weill Cornell Medicine, New York, NY USA; 6 Department of Surgery, Brigham and Women’s Hospital and Dana Farber Cancer Institute, Boston, MA USA; 7 Department of Pathology, Memorial Sloan-Kettering Cancer Center, New York, NY USA; 8 Department of Pathology, AC Camargo Cancer Center, Sao Paulo, Brazil; 9 Department of Pathology, GZA-ZNA, Antwerp, Belgium; 10 Division of Research, Peter Mac Callum Cancer Centre, Melbourne, Australia; 11 Department of Pathology, Institut Jules Bordet, Brussels, Belgium; 12 Translational Breast Cancer Laboratory, Institut Jules Bordet, Brussels, Belgium; 13 Department of Pathology and Surgery, University of Ottawa, Ottawa, Canada; 14 Department of Medicine, Yale University School of Medicine, New Haven, CT USA

**Keywords:** Predictive markers, Breast cancer

## Abstract

There is a clinical need to predict sensitivity of metastatic hormone receptor-positive and HER2-negative (HR+/HER2−) breast cancer to endocrine therapy, and targeted RNA sequencing (RNAseq) offers diagnostic potential to measure both transcriptional activity and functional mutation. We developed the SET_ER/PR_ index to measure gene expression microarray probe sets that were correlated with hormone receptors (*ESR1* and *PGR*) and robust to preanalytical and analytical influences. We tested SET_ER/PR_ index in biopsies of metastastic HR+/HER2− breast cancer against the treatment outcomes in 140 patients. Then we customized the SET_ER/PR_ assay to measure 18 informative, 10 reference transcripts, and sequence the ligand-binding domain (LBD) of *ESR1* using droplet-based targeted RNAseq, and tested that in residual RNA from 53 patients. Higher SET_ER/PR_ index in metastatic samples predicted longer PFS and OS when patients received endocrine therapy as next treatment, even after adjustment for clinical-pathologic risk factors (PFS: HR 0.534, 95% CI 0.299 to 0.955, *p* = 0.035; OS: HR 0.315, 95% CI 0.157 to 0.631, *p* = 0.001). Mutated *ESR1* LBD was detected in 8/53 (15%) of metastases, involving 1−98% of *ESR1* transcripts (all had high SET_ER/PR_ index). A signature based on probe sets with good preanalytical and analytical performance facilitated our customization of an accurate targeted RNAseq assay to measure both phenotype and genotype of ER-related transcription. Elevated SET_ER/PR_ was associated with prolonged sensitivity to endocrine therapy in patients with metastatic HR+/HER2− breast cancer, especially in the absence of mutated *ESR1* transcript.

## Introduction

Endocrine therapy is the principal treatment for metastatic HR+/HER2− breast cancer until resistance becomes clinically manifest.^1,2^ Molecular progression from reliance on estrogen is generally accepted as the basis of acquired resistance, and this can sometimes be identified as reduced hormone receptor expression (ER and PR loss in approximately 10% and 20%, respectively, at first metastatic relapse^3–6^), upregulation of alternative growth pathways, acquisition of constitutively activating gene mutations in the ligand-binding domain (LBD) sequence of *ESR1*,^7,8^ or acquisition of other aberrations that accelerate growth and promote survival. Notably, the onset, rate and mechanisms of molecular progression vary for each patient.

Clinically, endocrine treatment resistance is recognized from short disease-free interval in the adjuvant or metastatic setting of endocrine treatment, development of visceral disease, or loss of ER or PR in metastatic breast cancer. However, these criteria are inexact. A quantitative biomarker of sensitivity to endocrine therapy (SET) in metastatic cancer might potentially contribute clinically useful information to address a clinical conundrum: whether to continue with endocrine therapy,^9^ combine this with another targeted therapy, or switch to chemotherapy-based treatment. Furthermore, it might inform a secondary concern: when in the course of therapies for metastatic breast cancer it might be optimal to add a cdk4/6 or PI3kinase/mTOR inhibitor to endocrine therapy. For example, it is still unclear whether addition of currently approved targeted agents to endocrine therapy in advanced disease improves progression-free survival (PFS) by reversing endocrine resistance or augmenting partial endocrine sensitivity.

Based on our previous development of a signature of *ESR1*-related transcripts in early breast cancer,^10^ we hypothesized that a combination of genes with expression related to both estrogen and progesterone receptors (gene symbols *ESR1* and *PGR*), but not proliferation, might predict sensitivity to endocrine therapy in metastatic breast cancer.^11^ We also considered preanalytical and analytical effects on measurement of gene expression in our approach to select transcripts for our signature, in order to develop a technically robust signature of a few genes that we could then translate to a customized assay with strong analytical validity. In addition, we considered that elevated hormone receptor-related transcription might represent natural activity (and indicate sensitivity to endocrine therapy) or perversely result from constitutive activating mutation of *ESR1* transcripts (already implicated in resistance to aromatase inhibitors^12^). Overall, we felt that the current evidence for altered biology of progressive breast cancer after relapse requires a more specialized approach to risk stratification than adoption of multi-gene assays that were developed for the earliest stages of hormone receptor-positive breast cancer.^13–15^ Hence, we aimed to combine both genotypic and phenotypic information, using a customized RNA sequencing (RNAseq) assay to measure sensitivity to endocrine therapy (SET).

## Results

### Definition of the SET_ER/PR_ index

**Fig. 1:**
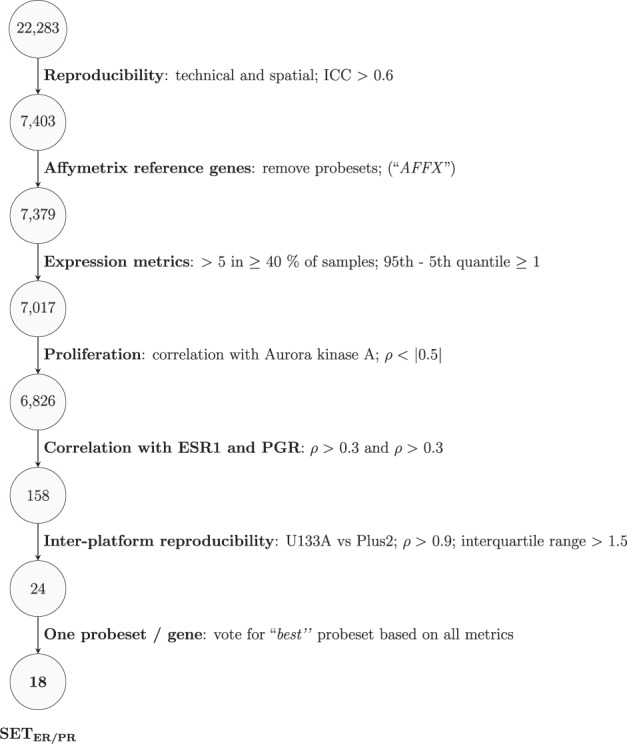
Feature selection process. We filtered probe sets based on performance in studies on technical and spatial reproducibility (i.e. intratumoral heterogeneity), association with ESR1 and PGR expression and association with proliferation. We applied additional filtering steps to reduce the signature to 18 robust probe sets

Eighteen informative transcripts (correlated with both *ESR1* and *PGR* and without obvious association with proliferation) and ten reference transcripts were selected for inclusion in the SET_ER/PR_ index (Fig. 1, Supplementary Table 2). The reference genes were selected based on minimal variability and high reproducibility across 331 hormone receptor-positive, HER2-negative samples of the training set (Supplementary Fig. 1). SET_ER/PR_ was defined as: ${\mathrm {SET}}_{{\mathrm {ER}}/{\mathrm {PR}}} = \frac{{\mathop {\sum }\nolimits_{i = 1}^{18} T_i}}{{18}} - \frac{{\mathop {\sum }\nolimits_{j = 1}^{10} R_j}}{{10}} + 2$, where *T*_*i*_ is the expression of the *i*th of the 18 informative genes and *R*_*j*_ the expression of the *j*th of the ten reference genes. The distribution of SET_ER/PR_ index scores was scaled to be above zero for most HR+/HER2− cancers and below zero for HR− cancers. Negative score values are assigned zero value to avoid confusion and variance from low expression of the target genes. We used the median value of SET_ER/PR_ in the clinically annotated dataset as a cut-off value to assign patients to groups with high vs. low SET_ER/PR_.

### Performance under preanalytical and analytical conditions used for development

SET_ER/PR_ was robust to technical replication (ICC = 0.990), intratumoral sampling (ICC = 0.953), type of cancer sample (cytology vs. tissue, *ρ*_P_ = 0.952), and type of microarray platform (U133A vs. Plus2.0 arrays, *ρ*_P_ = 0.990). Score values obtained from Plus2.0 arrays had a slight bias towards higher values when compared to U133A microarrays (Supplementary Fig. 2).

### Performance under independent preanalytical and analytical conditions

Supplementary Fig. 3 demonstrates the performance of SET_ER/PR_ in preanalytical and analytical validation studies that were not previously used in the feature selection process. The cross-platform reproducibility was validated in an independent dataset of 32 cases profiled on both U133A and Plus2.0 microarrays with *ρ*_P_ = 0.994 for the corrected score and *ρ*_P_ = 0.995 for inter-laboratory reproducibility. The technical reproducibility of the assay on U133A microarrays was validated in an independent dataset of 63 data pairs (*ρ*_P_ = 0.994). SET_ER/PR_ was stable over relevant ranges of contamination with liver or normal breast tissue with negative score values regressing more rapidly to the baseline levels from normal liver or normal breast tissues. Categories of high vs. low SET_ER/PR_ index (relative to median of 0.82) were consistent (*κ* = 0.881 and 0.905, respectively) over a range of 0−90% RNA added from normal liver or breast. There was no statistically significant effect of time delay (ex vivo ischemic time) and sample preservation method (RNAlater versus snap frozen) on SET_ER/PR_ measurements (Supplementary Table 3).

### Prognostic performance in metastatic breast cancer

The characteristics of 140 patients with hormone receptor-positive, HER2-negative metastatic breast cancers are summarized in Table 1. The observed range of SET_ER/PR_ was comparable in samples from different sites of metastasis (Supplementary Fig. 4). SET_ER/PR_ was positively associated with PR immunohistochemical status (*p* < 0.0001) and prior clinical history of endocrine sensitivity (*p* = 0.0471, Supplementary Fig. 4), and negatively associated with the number of prior progression events (*p* = 0.009).

**Table 1:** Patient characteristics

Stage at initial diagnosis		
Stage IV	45	32
Stage I−III	95	68
Visceral metastases		
Yes	80	57
No	60	43
Progesterone Receptor Status (Immunohistochemistry)		
Positive	80	57
Negative	60	43
Prior sensitivity		
Sensitive	70	50
Resistant	39	28
No prior endocrine therapy	31	22
Number of events biopsied		
Initial diagnosis	20	14
1st	42	30
2nd	26	19
3rd	14	10
4th or more	38	27
Treatment		
Endocrine	97	69
Chemotherapy	33	24
Other	8	6
Radiotherapy alone	2	1
	**Median**	**Range**
Age		
Years	55	32−82
Progression-free survival		
Months	5.53	0.16−74
Overall survival		
Months	24	0.16−126

Characteristics of the 140 patients with stage IV breast cancer

**Fig. 2:**
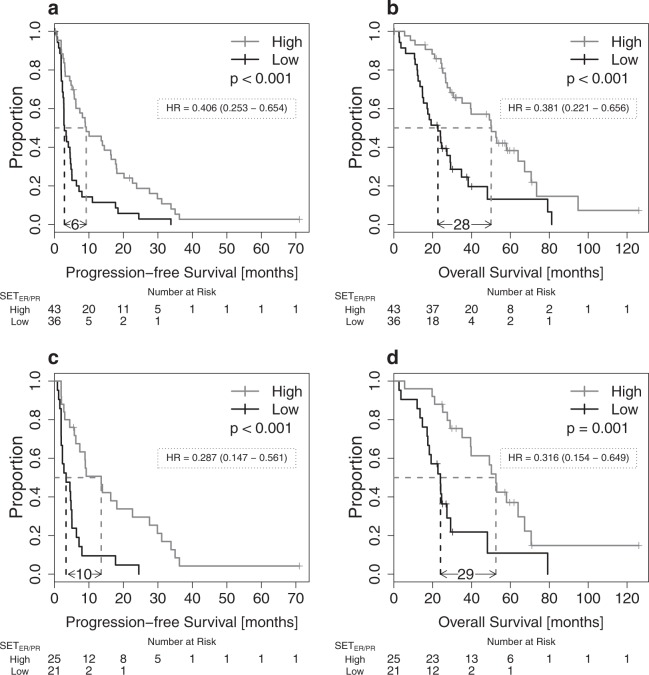
SET_ER/PR_ and patient survival. SET_ER/PR_ and progression-free and overall survival in patients with HR+/HER2 metastatic breast cancer. Kaplan−Meier curves are shown for progression-free **a** and overall survival **b** in patients that presented with relapsed stage IV breast cancer and received endocrine therapy as next treatment and for the clinically relevant subgroup of patients with a prior history of sensitivity to adjuvant or metastatic endocrine treatment **c**, **d**

The continuous SET_ER/PR_ index was prognostic for PFS and OS in patients receiving endocrine-based therapy (PFS: hazard ratio (HR) 0.51 (0.41−0.74), *p* < 0.001; OS: 0.40 (0.26–0.62), but not in patients receiving chemotherapy (PFS: HR 0.76 (0.45−1.27), *p* = 0.290). We selected the median value (0.82) as threshold to dichotomize SET_ER/PR_ index. Dichotomized SET_ER/PR_ was independently prognostic for PFS (Table 2) and OS (Table 3) in univariate and multivariate analyses with standard clinical-pathologic risk factors. We further analyzed the survival of patients whose biopsy was obtained at a time of recurrence (after prior systemic therapy) and whose next treatment included endocrine therapy. In patients who had previously demonstrated clinical evidence of sensitivity to endocrine therapy, the continuous SET_ER/PR_ index was independently prognostic for PFS in a multivariate model that included PR immunohistochemistry status of the metastasis, the number of prior relapse events, and the presence or absence of any visceral metastasis (Tables 2 and 3). Figure 2 shows Kaplan−Meier plots using the dichotomized SET_ER/PR_ index in the same cohort of patients. SET_ER/PR_ was significantly associated with patient outcome over a wide range of different possible cut-points (Supplementary Fig. 4D).

**Table 2:** SET_ER/PR_ for prediction of progression-free survival

	HR	95 % CI	*p*
Chemotherapy (*N* = 33)			
SET_ER/PR_	0.935	0.426−2.053	0.868
Endocrine treatment (*N* = 97)			
SET_ER/PR_	0.420	0.273−0.644	<0.001
Endocrine treatment and relapsed stage IV (*N* = 79)			
SET_ER/PR_	0.407	0.253−0.654	<0.001
Endocrine treatment and relapsed stage IV			
SET_ER/PR_	0.534	0.299−0.955	0.035
PR status	0.604	0.335−1.087	0.093
Visc. met.	1.502	0.851−2.653	0.161
Event >2	2.904	1.457−5.788	0.002
Prior Sens.	0.466	0.246−0.884	0.019
Endocrine treatment and relapsed stage IV and prior sensitivity (*N* = 46)			
SET_ER/PR_	0.287	0.147−0.561	<0.001
Endocrine treatment and relapsed stage IV and prior sensitivity			
SET_ER/PR_	0.303	0.143−0.642	0.002
PR status	0.497	0.249−0.992	0.047
Visc. met.	1.063	0.509−2.220	0.871
Event >2	3.779	1.699−8.407	0.001

Cox regression analyses for prediction of progression-free survival using the dichotomized SET_ER/PR_. Results are shown for patients that received chemotherapy and those that received endocrine treatment. Uni- and multivariate analyses are shown for the clinically relevant subgroups of patients that received endocrine treatment and presented with relapsed stage IV disease and the subset of patients with a prior history of endocrine sensitivity

*HR* hazard ratio, *CI* confidence interval

**Table 3:** SET_ER/PR_ for prediction of overall survival

	HR	95 % CI	*p*
Chemotherapy (*N* = 33)			
SET_ER/PR_	0.813	0.318−2.077	0.666
Endocrine treatment (*N* = 97)			
SET_ER/PR_	0.391	0.239−0.638	<0.001
Endocrine treatment and relapsed stage IV (*N* = 79)			
SET_ER/PR_	0.381	0.221−0.656	0.001
Endocrine treatment and relapsed stage IV			
SET_ER/PR_	0.315	0.157−0.631	0.001
PR status	0.524	0.267−1.029	0.061
Visc. met.	1.808	0.945−3.460	0.074
Event >2	4.463	1.943−10.25	<0.001
Prior Sens.	0.331	0.156−0.700	0.004
Endocrine treatment and relapsed stage IV and prior sensitivity (*N* = 46)			
SET_ER/PR_	0.316	0.154−0.649	0.002
Endocrine treatment and relapsed stage IV and prior sensitivity			
SET_ER/PR_	0.275	0.119−0.637	0.003
PR status	0.433	0.189−0.995	0.049
Visc. met.	1.827	0.785−4.250	0.162
Event >2	5.222	2.082−13.10	<0.001

Cox regression analyses for prediction of overall survival using the dichotomized SET_ER/PR_. Results are shown for patients that received chemotherapy and those that received endocrine treatment. Uni- and multivariate analyses are shown for the clinically relevant subgroups of patients that received endocrine treatment and presented with relapsed stage IV disease and the subset of patients with a prior history of endocrine sensitivity

*HR* hazard ratio, *CI* confidence interval

In addition to the multivariate analyses using standard clinical and pathological tumor characteristics, we evaluated if *AURKA* as marker of proliferation might add prognostic information. As illustrated in Supplementary Table 4, *AURKA* is prognostic for both PFS and OS in patients who received chemotherapy as next treatment, independent of SET_ER/PR_, and also after adjustment for clinical and pathological characteristics. If patients received endocrine therapy as next treatment, expression of *AURKA* did not add prognostic information when SET_ER/PR_ was included in bivariate and multivariate models, while SET_ER/PR_ retained its significance.

### Customization of the SET_ER/PR_ assay using targeted RNA sequencing (RNAseq)

The customized RNAseq assay integrates measurements of ER and PR-related transcriptional activity (SET_ER/PR_ index) and the proportion of *ESR1* transcript reads with activating LBD mutation. SET_ER/PR_ index was calibrated between microarray and customized RNAseq assays in 40 breast cancer samples analyzed in duplicate with both assays (Supplementary Fig. 5). There was excellent interassay agreement (*ρ*_P_ = 0.965 and *κ* = 0.823) in an independent test of 23 breast cancer samples.

### Proportion of *ESR1* transcript reads with LBD mutation related to the SET_ER/PR_ index

**Fig. 3:**
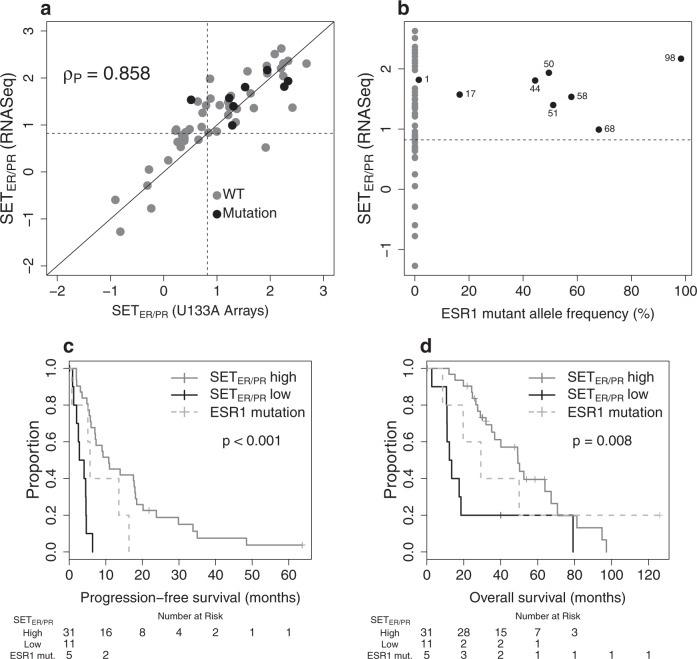
Customized RNA-seq. SET_ER/PR_ assay. For a subset of cases, SET_ER/PR_ measurements were repeated on the RNA-seq. platform using leftover RNA of the clinically annotated dataset. **a** The RBA-seq. assay was correlated with the U133A measurements with a good reproducibility of the cut-point (dotted lines). Of note, the cases with ESR1 mutations (black dots) have higher SET_ER/PR_ values. **b** Gene expression measurements are plotted against the observed allele frequency of ESR1 mutations (the numbers represent % ESR1 LBD reads with mutation). **c**, **d** Kaplan−Meier plots for patients with HR+/HER2− metastatic disease that received endocrine therapy as next treatment

The customized RNAseq assay detected mutations in the LBD of *ESR1* in 8/53 samples, with an average of 33,000-fold coverage depth. Metastases with an *ESR1* mutation had high SET _ER/PR_ index (Fig. 3). We confirmed that the customized RNAseq assay for SET_ER/PR_ index achieved a similar prognostic separation (Fig. 3) to the original microarray assay (Fig. 2) in patients treated with endocrine therapy. An exploratory analysis suggested that the prognosis among patients with an *ESR1* LBD mutation (and consequently higher SET_ER/PR_ index) may be intermediate between those with low SET_ER/PR_ index and high SET_ER/PR_ index with wild-type *ESR1* (Fig. 3).

## Discussion

SET_ER/PR_ index is an unbiased calculation based on the straightforward concept of measuring transcription associated with *ESR1* and *PGR* expression, which avoids over-fitting from modeling on outcome data. The assay was robust to critical preanalytical conditions (tissue and cytologic samples, ex vivo ischemia, preservation or fixation of tissue samples, and intratumoral spatial heterogeneity) and analytical conditions (technical reproducibility at all levels of the assay procedure, different technical platforms for the assay). We also describe how it was customized into an assay that also integrates measurement of mutated *ESR1* transcripts.

To our knowledge, SET_ER/PR_ is the first multigene expression assay to be developed specifically for metastatic breast cancer. Higher SET_ER/PR_ index was associated with longer PFS and OS for patients treated by endocrine therapy, particularly for those who had previously demonstrated clinical sensitivity to hormonal therapy. Although we observed that SET_ER/PR_ was not associated with outcome in patients treated with chemotherapy, that cohort was too small to be able to make any conclusion. Additionally, the observation might be confounded because chemotherapy is usually offered when there is already clinical evidence for endocrine resistance. We also note that high expression of *SLC39A6* is observed in the SET_ER/PR_ index (Supplementary Fig. 1, Supplementary Table 2). This transcript encodes LIV-1, the membrane target for the antibody-drug conjugate SGN-LIV1.^16^

*ESR1* mutations occur within the LBD sequence, and are rare in primary cancer. They commonly occur in relapsed metastatic disease, and are possibly more frequent after treatment with aromatase inhibitors.^7,8,17^ These mutations induce constitutive receptor activity and have been identified as a mechanism of resistance to estrogen-depriving therapies, while patients might still benefit from selective estrogen receptor degradation (SERD) treatment, for example fulvestrant. In the FERGI and PALOMA-3 trials, *ESR1* mutations had no effect on PFS in patients receiving fulvestrant with or without a PI3K inhibition or cdk4/6 inhibition, respectively.^18,19^ In the BOLERO-2 trial, patients with *ESR1* mutations had shorter PFS under exemestane with or without everolimus.^20^ In the SoFEA trial, patients with *ESR1* mutations had a longer PFS after a regimen containing fulvestrant as compared to anastrozole.^19^ While available data are inconclusive, there appears to be a trend toward associations of *ESR1* mutation and endocrine resistance that might be reversed by SERD treatment.

We observed that *ESR1* mutations were associated with higher values of SET_ER/PR_ (presumably because these mutations are constitutively activating), but only some cancers with higher values of SET_ER/PR_ index contained an *ESR1* mutation. Indeed, we observed three main groups in our data: (1) high SET_ER/PR_ index with wild-type *ESR1* (better prognosis with endocrine therapy); (2) low SET_ER/PR_ index with wild-type *ESR1* (worse prognosis with endocrine therapy); and (3) high SET_ER/PR_ index with activating mutation of *ESR1* (possibly intermediate prognosis with endocrine therapy). This potentially highlights the importance of integrating both transcriptional measurements (phenotype) with mutation status (genotype) to understand genomic effects on sensitivity to endocrine therapy. A future challenge will involve accurate combination of SET_ER/PR_ index with the percent of mutated *ESR1* transcripts, since this RNAseq assay precisely measures that fraction even to minimal values because the number of transcripts per cell and the depth of sequencing are both high. Our results are in line with the assumption that transcriptional activation by ER in metastatic disease could be pertinent to endocrine sensitivity in the context of wild-type *ESR1*, but could be active yet resistant to endocrine treatment if predominantly due to mutant *ESR1*. However, a far larger experience of samples data and treatment outcomes will be necessary to understand whether this is a real observation and whether the proportion of mutant *ESR1* is relevant to outcomes. Clinical utility will depend on further clinical validation and how the information might inform treatment options.

It was important to include genes with expression related to *PGR* expression. The presence of progesterone receptor is considered an indicator of estrogen-dependence and better differentiation of a tumor with the most favorable prognosis being associated with the phenotype or ER- and PR-expressing tumors.^21^ Recently, it has been shown that PR can directly remodulate ER-associated transcriptional profiles by altering its chromatin-binding characteristics, indicating complex interaction between ER and PR.^22^ In early-stage breast cancer, estrogen receptor-related transcriptional profiles can predict prognosis following endocrine therapy^10^ and PR might be prognostic rather than predictive for endocrine response.^23^ However, PR holds greater interest for endocrine prediction in stage IV disease.^11^ Thus, even if we did not have semiquantitative data on PR expression for all patients, it is important that the SET_ER/PR_ index remained prognostic for endocrine therapy even after adjustment for PR immunohistochemistry status (≥10% nuclear staining) and the other relevant clinical risk factors (Table 2).

Metastatic breast cancer is a dynamic disease, prone to heterogeneity and evolving over time and under the selective pressure of different treatments.^24,25^ At this time the AURORA initiatives are aiming to characterize the molecular progression of metastatic breast cancer based on next-generation sequencing using serial biopsies taken over the course of the disease.^25^ This might lead to further insight into molecular evolution. Indeed, we don’t know yet whether the SET_ER/PR_ index would change during successive progression events or in response to different classes of treatment.

Treatment of stage IV HR+/HER2− breast cancer typically relies on available endocrine treatments^9,26^ until more rapidly progressive disease favors a switch to chemotherapy^2,27^ However, this treatment strategy increasingly requires nuanced clinical judgment, as the selection of treatment options continues to expand to include additional endocrine agents, alone or combined with targeted molecular agents, chemotherapy, and other molecularly targeted approaches. So an index of tumoral sensitivity to endocrine therapy might become a clinically useful metric, alone or in combination with proven biomarkers to select among the other treatment alternatives.^28^ In this context, the SET_ER/PR_ index might inform the selection of next treatment: switch endocrine therapy, augment endocrine therapy with a targeted molecular therapy (such as mTOR, PI3K, or cdk4/6 inhibition), include an SERD agent to target emergent mutated *ESR1* clone, or switch to a different treatment strategy (such as chemotherapy, immune therapy). Of course, any definitive statement on such clinical utility would require testing the SET_ER/PR_ index using samples from randomized trials and goes beyond the scope of this first description of the assay. But even within those trials, we might gain insight as to whether the addition of different targeted therapies might augment sensitivity, or reverse resistance to endocrine therapy—questions that are difficult to answer without a biomarker for endocrine sensitivity.

There are several important caveats to the interpretation and generalizability of our results. Despite an overall sample size of 140 prospective biopsies of relapsed metastatic disease, the clinical and treatment subsets are small, requiring cautious interpretation of these results. This is a limitation of the combined analysis of SET_ER/PR_ index and percent mutated *ESR1* transcripts. Another limitation is the lack of an independent clinically annotated cohort to validate the findings that would also allow the definition and validation of an optimized cut-point for patient stratification.

Overall, this manuscript introduces a novel approach to assay development and this assay appears to be analytically valid. The promising clinical performance is still exploratory, and further independent clinical validation studies of the assay and its cut-point will still be required.

## Methods

All patients gave informed written consent to take part in the study and for the use of tissue material for research purposes. Protocols were approved by the MD Anderson Institutional Review Board (IRB). The microarray and accompanying data are available on NCBI GEO and summarized under a figshare metadata record.^29^

### Discovery cohort

The discovery cohort of Affymetrix U133A microarrays (*N* = 389) from invasive hormone receptor-positive breast cancers included 242 cases from our published dataset^10^ and 147 additional samples (GSE129551), all derived from fresh tissue or FNA biopsy samples obtained prior to any systemic therapy and stored frozen at −80 °C in *RNAlater* (approved IRB protocols LAB99-402, LAB04-0093). Receptor status, tumor stage and type of tumor samples are described in Supplementary Table 1.

ER- and PR-positivity was defined as nuclear immunostaining in ≥10% of tumor cells. Antibody clones 6F11, dilution 1:35, and PGR1294, dilution 1:200, were used on a Leica Bond-Max instrument according to standard procedures. HER2-positivity was defined as immunohistochemistry score of 3+ membrane staining and/or gene amplification (HER2/CEP17 ratio >2.2) by fluorescence in situ hybridization.

### Gene expression profiling for target and reference transcripts

RNA was extracted, processed and hybridized to Affymetrix human genome U133A microarrays (U133A GeneChip, Affymetrix, Santa Clara, CA, USA) as described previously. In brief, the raw intensity files were processed using the MAS5.0 algorithm to generate probe set-level intensities, normalized to a median array intensity of 600, log2-transformed and scaled using the expression of 1322 breast cancer reference genes within each sample.^10,30^ Target probe sets for gene transcripts in the 389 cases of the discovery cohort were identified based on Spearman’s rank correlation coefficient for coexpression with *ESR1* and *PGR* (probe sets 205225_at and 208305_at) in hormone receptor-positive breast cancer samples. Reference probe sets were selected based on consistency and range of expression values. This manuscript follows REMARK guidelines.^31^

### Studies of preanalytical and analytical robustness

We conducted a series of studies to evaluate the reproducibility of gene expression measurements in breast cancer samples according to replication of technical, intratumoral, interplatform, and inter-sample type conditions (IRB protocols LAB08-0823, LAB08-0824). These included 6 technical (analytical) replicates from 20 breast cancers (GSE129558), 3 tumor samples from each of 51 breast cancers (GSE129557), inter-sample type comparisons of 116 matched cytology and tissue samples (GSE129559) that were collected from multiple institutions, and interplatform comparisons of Affymetrix U133A and Plus2 array platforms from 88 breast cancers (GSE129556). Figure 1 provides an overview of how these studies were used to select the probe sets for the final gene signature. We tested the robustness of the final SET_ER/PR_ gene expression index in other studies: 11 breast cancers contaminated with increasing known amounts of liver RNA (GSE33116); 10 other breast cancers diluted with increasing known amounts of normal breast RNA (GSE124648); 17 other breast cancers with increasing duration of ischemic delay at room temperature, testing two sample preservation methods (GSE25011)^32^; matched U133A and Plus2 arrays in two different laboratories (MDACC and JBI; GSE17700); and technical replicates using U133A arrays in another 63 breast cancers from MDACC (GSE129560).

### Development of customized RNAseq assay

We employed a digital PCR-based RNAseq strategy with three steps: (1) droplet-generation using RainDance Source system (BioRad, Hercules, CA) and one-step RT-PCR reaction (first PCR) to target the regions of interest with our custom multiplex primer set; (2) second PCR to incorporate RainDance DirecSeq primers for sample indexing and Illumina specific adapters for cluster generation/sequencing; (3) library quantification, QC, and llumina MiSeq sequencing (Illumina, San Diego, CA). We perform pooled sequencing of up to 40 sequence libraries per flow cell. The read count of each targeted sequence was log2 transformed, and the sequence reads of the LBD of the *ESR1* transcript were analyzed for single nucleotide variants and reported as the percent of *ESR1* reads and type of mutation.

### Clinical cohort with stage IV breast cancer

Patients with metastatic HR+ breast cancer were offered participation in a prospective research protocol to obtain a research sample at the time of their clinical biopsy of metastasis at MD Anderson (protocol LAB04-0093) between 2004 and 2013, obtained as fine-needle aspiration (FNA) or core biopsy (CBX). Their next treatment was recorded and was at the discretion of their oncologist. A total of 234 samples were profiled using Affymetrix U133A gene expression microarrays, 212 microarrays passed our quality control analysis. We excluded 32 HER2-positive and 26 hormone receptor-negative cases based on immunohistochemistry and (where appropriate) HER2 in situ hybridization testing of the metastatic samples. Fourteen additional cases were excluded for other reasons (no follow-up data after biopsy, diagnosis other than breast cancer), resulting in 140 eligible cases with quality microarray data in this study (GSE124647). Median PFS and OS were 5.5 and 24.0 months, respectively (Table 1). PR positivity was defined as ≥10% nuclear immunostaining. Proliferation (Ki-67 immunohistochemistry) is not usually assessed in metastatic samples, so we evaluated Aurora kinase-A (AURKA; probe set 208079_s_at) as a reliable genomic marker for proliferation in multivariate survival analyses.^33^ The clinical variable of prior endocrine sensitivity was defined as a history of at least 6 months of freedom from progression while on endocrine therapy for metastatic disease or 5 years adjuvant endocrine therapy for primary breast cancer without recurrence. A subset of 53 cases was available for analysis of *ESR1* gene mutations by RNAseq.

### Statistical methods

Pearson’s correlation coefficient (*ρ*_P_) was used to compare cross-platform and cross-tissue reproducibility of each candidate probe set on the array. The intraclass correlation coefficient (ICC) was used to evaluate intra-assay and intratumoral reproducibility. A linear mixed-effects model (LME) with random within-group intercept was used to estimate the effect of sample preservation method (RNA*later* vs. fresh frozen) and time delay (0 vs. 40 min)^32^ using the r package *lme4*. The effect of sample stabilization delay (cold ischemic time) was assessed using a similar model with fixed slope (for the cold ischemic time effect) and random intercept (for biological variation among tumors). The statistical significance of the coefficients was evaluated by using the likelihood ratio test to compare the full model with a reduced model that did not include the term of interest. To examine the impact of contamination with normal breast tissue and liver tissue, SET_ER/PR_ values were plotted against the percentage of contaminant. Fleiss’ *κ* statistic for multiple raters was used to evaluate the reproducibility of risk class assignment. We used the R package *survival* for survival analyses. PFS was defined as the time from the start of new treatment after the biopsy of relapsed disease, until disease progression or death from any cause. The endpoint definition for overall survival was death from any cause. We used Cox regression to model relationship between the continuous SET_ER/PR_ and survival outcomes. The Kaplan−Meier method and log-rank test were used to evaluate survival outcomes using the dichotomized score. All statistical analyses and computations were performed in R v. 3.1.2 ^34^ and Bioconductor.^35^

### Reporting summary

Further information on experimental design is available in the Nature Research Reporting Summary linked to this paper.

## Supplementary information

Supplementary Information

Reporting Summary Checklist

## Data Availability

The data generated and analyzed during this study are described in the following metadata record: 10.6084/m9.figshare.7998809.^29^ Datasets are available on NCBI Gene Expression Omnibus (GEO) summarized under SuperSeries GSE124648.

## Supplementary information

**Supplementary information** accompanies the paper on the *npj Breast Cancer* website (10.1038/s41523-019-0111-0).
