# Supplementary material for: SETER/PR: a robust 18-gene predictor for sensitivity to endocrine therapy for metastatic breast cancer
Source: NPJ Breast Cancer. 2019 May 30;5:16. doi: 10.1038/s41523-019-0111-0 (PMC6542807; doi:10.1038/s41523-019-0111-0)
Supplement: Supplementary file 1 — Supplementary Information [file 41523_2019_111_MOESM1_ESM.pdf]

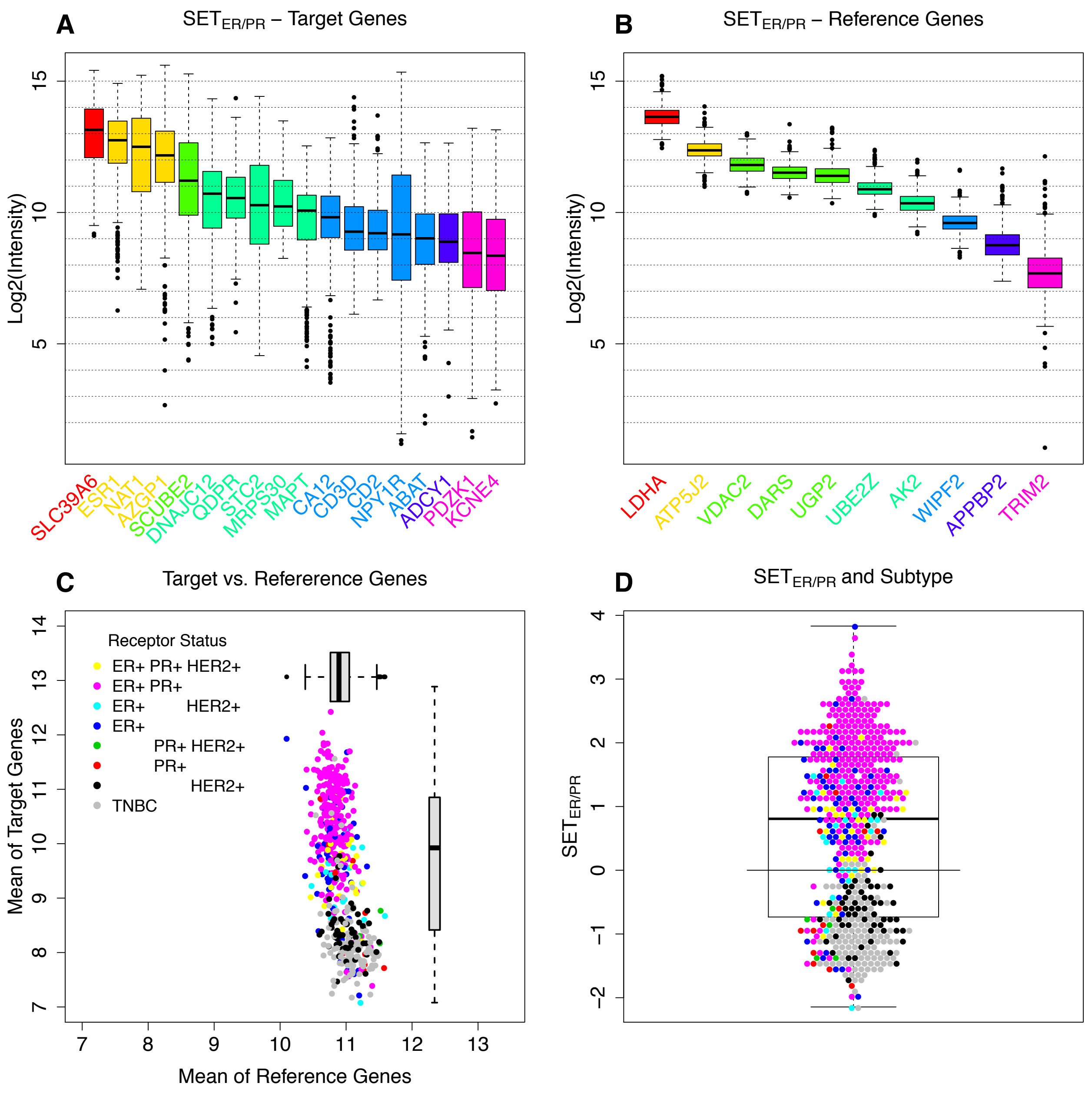

Supplementary Figure 1 Distribution of the ESR1- and PGR-associated genes in the hormone-receptor-positive discovery cohort (A) and the reference genes (B). The reference genes cover the whole range of gene expression (colors). (C) Distribution of the target- and reference genes in the discovery dataset. The mean of the target genes is plotted against the mean of the reference genes. (D) Using 175 additional hormone receptor-negative cases (GSE25066), the score was scaled linearly to assign negative values to hormone receptor-negative tumors (as defined by immunohistochemistry). The boxes show the median, upper and lower quartiles of the data, the whiskers extend to the most extreme data point which is no more than 1.5 times the interquartile range from the box.

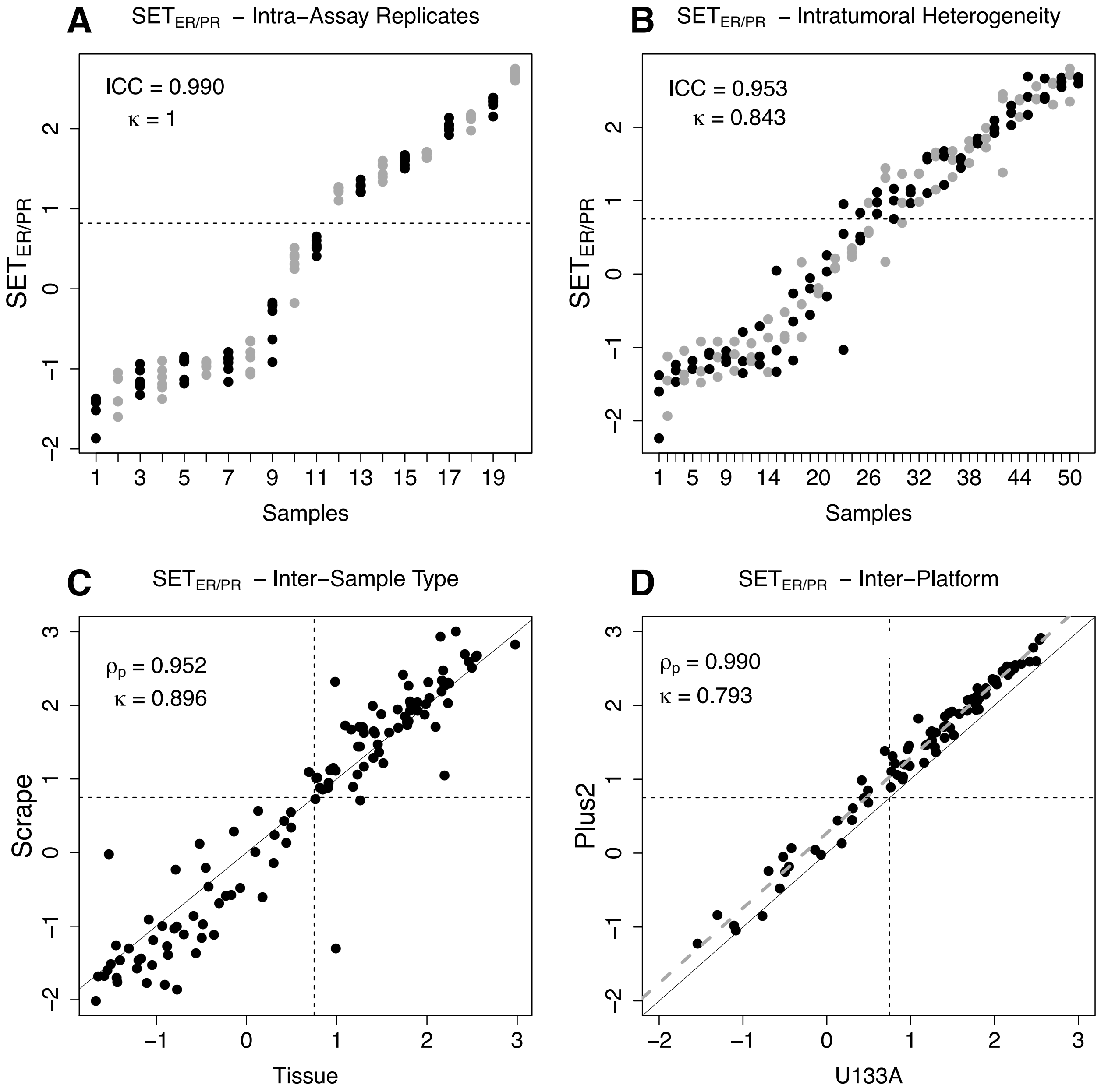

Supplementary Figure 2 Performance of $SET_{ER/PR}$ in analytical datasets used for development. $SET_{ER/PR}$ had an excellent reproducibility in six technical replicates (A) and three intra-tumoral replicates (B). The individual measurements for each sample are plotted above each other and samples are ordered according to mean score values. Reproducibility across different tissue samples was excellent (C). Score values obtained from Plus2.0 arrays had a slight bias towards higher values as compared to U133A microarrays (D).

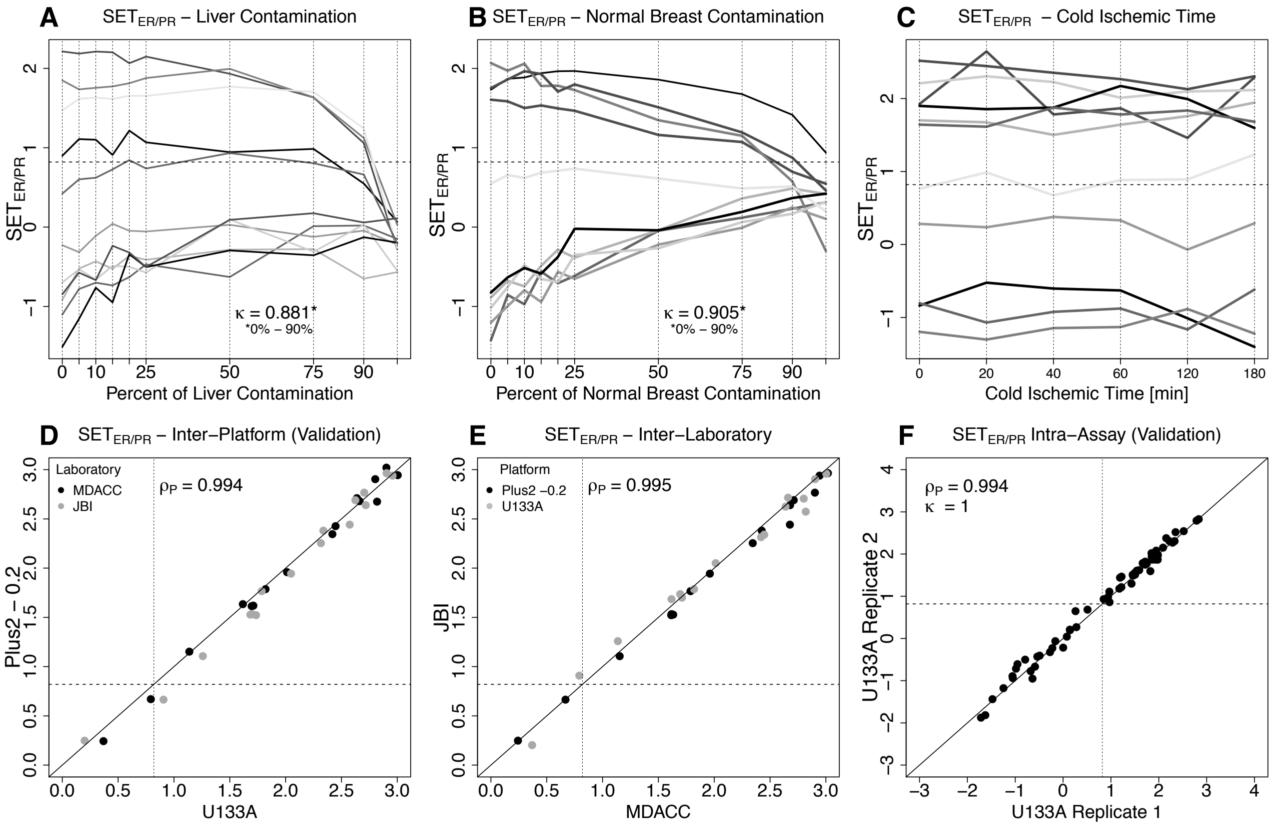

Supplementary Figure 3 Performance of SET$_{ER/PR}$ in independent datasets. SET$_{ER/PR}$ was stable over relevant ranges of contamination with liver (A) or normal breast tissue (B). There was no statistically significant effect of cold ischemic delay and sample preservation method (C). The cross- platform reproducibility was validated in an independent dataset of 32 cases profiled on both U133A and Plus2.0 microarrays (D) and in two different laboratories (E). The technical reproducibility on U133A microarrays was validated in an independent dataset (N= 63; F).

Supplementary Table 1

		Discovery I	Discovery II	Total
Subtype				
	HR+/HER2-	204	28	232
	HR+/HER2+	38	19	57
AJCC Stage				
	I	4	20	24
	II	4	20	24
	III	127	80	207
	IV	1	1	2
	NA	0	26	26
Tissue type				
	Surgical	0	134	134
	Cytology	242	13	255

Supplementary Table 1 Sample characteristics of the discovery data set

Supplementary Table 2

Affy ID	Symbol	Name	Entrez ID
202089_s_at	SLC39A6	solute carrier family 39 (zinc transporter), member 6 (LIV-1)	25800
203438_at	STC2	stanniocalcin 2	8614
204508_s_at	CA12	carbonic anhydrase XII	771
205225_at	ESR1	estrogen receptor 1	2099
205380_at	PDZK1	PDZ domain containing 1	5174
205440_s_at	NPY1R	neuropeptide Y receptor Y1	4886
205831_at	CD2	CD2 molecule	914
206401_s_at	MAPT	microtubule-associated protein tau	4137
209123_at	QDPR	quinoid dihydropteridine reductase	5860
209309_at	AZGP1	alpha-2-glycoprotein 1, zinc-binding	563
209459_s_at	ABAT	4-aminobutyrate aminotransferase	18
213245_at	ADCY1	adenylate cyclase 1 (brain)	107
213539_at	CD3D	CD3d molecule, delta (CD3-TCR complex)	915
214440_at	NAT1	N-acetyltransferase 1 (arylamine N-acetyltransferase)	9
218398_at	MRPS30	mitochondrial ribosomal protein S30	10884
218976_at	DNAJC12	DnaJ (Hsp40) homolog, subfamily C, member 12	56521
219197_s_at	SCUBE2	signal peptide, CUB domain, EGF-like 2	57758
222379_at	KCNE4	potassium channel, voltage gated subfamily E regulatory beta subunit 4	23704
200650_s_at	LDHA	lactate dehydrogenase A	3939
202961_s_at	ATP5J2	ATP synthase, H+ transporting, mitochondrial Fo complex, subunit F2	9551
211662_s_at	VDAC2	voltage-dependent anion channel 2	7417
201623_s_at	DARS	aspartyl-tRNA synthetase	1615
205480_s_at	UGP2	UDP-glucose pyrophosphorylase 2	7360
217750_s_at	UBE2Z	ubiquitin-conjugating enzyme E2Z	65264
212175_s_at	AK2	adenylate kinase 2	204
212050_at	WIPF2	WAS/WASL interacting protein family, member 2	147179
202631_s_at	APPBP2	amyloid beta precursor protein (cytoplasmic tail) binding protein 2	10513
202342_s_at	TRIM2	tripartite motif containing 2	23321

Supplementary Table 2 List of target genes (above the horizontal line) and reference genes (below) used for the calculation of $SET_{ER/PR}$.

Supplementary Table 3 – Tissue stabilization method and time delay

		Estimate	95 % CI	p-value
Fixed effects				
	Intercept	0.989	0.133 - 1.842	NA
	Time delay	0.017	-0.116 - 0.139	0.854
	Stabilization	-0.080	-0.212 - 0.050	0.219
Random effects				
	Between-tumor SD	1.311	NA	NA
	Within-tumor SD	0.205	NA	NA
	ICC	0.972	NA	NA

Time delay

		Estimate	95 % CI	p-value
Fixed effects				
	Intercept	0.939	0.108 - 2.082	NA
	Cold ischemic time	-0.001	-0.001 - 0.001	0.468
Random effects				
	Between-tumor SD	1.280	NA	NA
	Within-tumor SD	0.213	NA	NA
	ICC	0.957	NA	NA

Supplementary Table 3 Mixed-effects analysis of the effect of tissue preservation method (snap frozen vs. RNAlater) and prolonged cold ischemic delay (0 vs. 40 min) on $SET_{ER/PR}$ measurements (top). Mixed-effects analysis of the effect of prolonged cold ischemic delay (0, 20, 40, 60, 120, 180 min) on $SET_{ER/PR}$ measurements (bottom).

Supplementary Table 4

		Progression-free survival				Overall survival				
		Univariate Cox Regression					Univariate Cox Regression			
		HR	95 % CI		p		HR	95 % CI		p
Chemo-therapy (N = 33)	AURKA	1.694	1.201	2.389	0.003	AURKA	1.509	1.031	2.208	0.034
		Bivariate Cox Regression					Bivariate Cox Regression			
		HR	95 % CI		p		HR	95 % CI		p
	$SET_{ER/PR}$	1.010	0.450	2.264	0.981	$SET_{ER/PR}$	0.83	0.322	2.14	0.700
	AURKA	1.695	1.20	2.394	0.003	AURKA	1.502	1.030	2.193	0.035
		Multivariate Cox Regression					Multivariate Cox Regression			
		HR	95 % CI		p		HR	95 % CI		p
	$SET_{ER/PR}$	1.176	0.503	2.749	0.709	$SET_{ER/PR}$	0.914	0.336	2.487	0.860
	AURKA	2.051	1.356	3.103	0.001	AURKA	2.357	1.384	4.016	0.002
	Visc. Met.	0.969	0.397	2.366	0.945	Visc. Met.	0.607	0.211	1.745	0.354
	Event > 2	2.891	1.172	7.129	0.021	Event > 2	6.427	1.847	22.362	0.003
Endocrine Treatment (N = 97)		Univariate Cox Regression					Univariate Cox Regression			
		HR	95 % CI		p		HR	95 % CI		p
	AURKA	1.207	0.993	1.468	0.059	AURKA	1.253	1.002	1.567	0.048
		Bivariate Cox Regression					Bivariate Cox Regression			
		HR	95 % CI		p		HR	95 % CI		p
	$SET_{ER/PR}$	0.437	0.283	0.675	< 0.001	$SET_{ER/PR}$	0.415	0.252	0.685	0.001
	AURKA	1.136	0.944	1.366	0.176	AURKA	1.16	0.936	1.438	0.175
		Multivariate Cox Regression					Multivariate Cox Regression			
		HR	95 % CI		p		HR	95 % CI		p
	$SET_{ER/PR}$	0.597	0.326	1.094	0.095	$SET_{ER/PR}$	0.374	0.180	0.779	0.009
	AURKA	1.247	0.965	1.612	0.091	AURKA	1.292	0.971	1.719	0.079
	PR Status	0.600	0.332	1.082	0.090	PR Status	0.502	0.252	1.001	0.050
	Visc. Met.	1.560	0.884	2.755	0.125	Visc. Met.	1.881	0.990	3.571	0.054
	Event > 2	3.356	1.663	6.772	0.001	Event > 2	4.973	2.168	11.408	< 0.001
	Prior Sens.	0.501	0.266	0.945	0.033	Prior Sens.	0.344	0.163	0.727	0.005

Supplementary Table 4 Cox regression analyses for prediction of progression-free and overall survival including AURKA gene expression as a marker for proliferation. Results are shown for patients that received chemotherapy and those that received endocrine treatment.

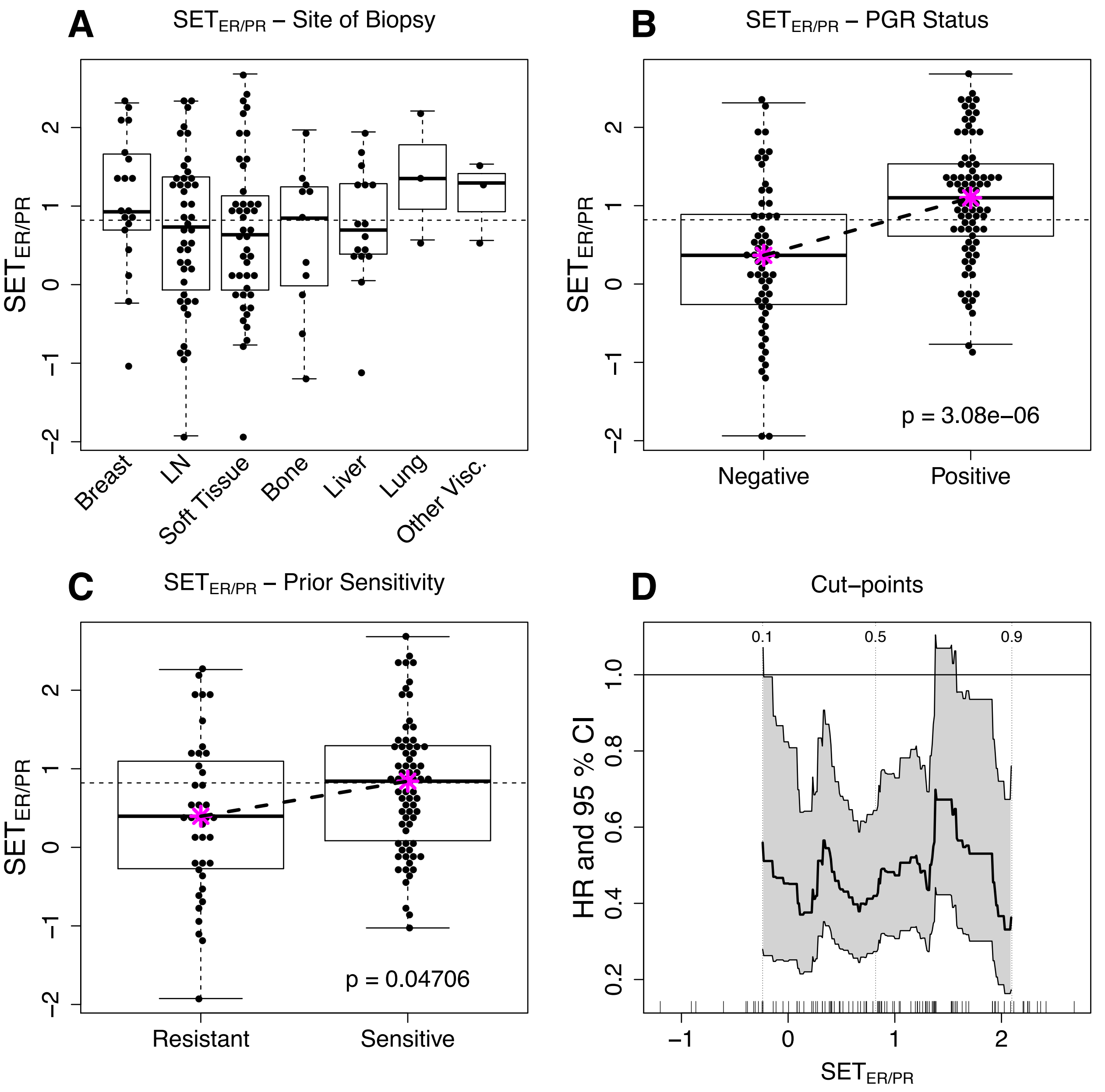

Supplementary Figure 4 *$SET_{ER/PR}$* and clinical and pathological tumor characteristics; cut-points. (A) Site of protocol biopsy, (B) PGR status by immunohistochemistry and (C) prior sensitivity to endocrine treatment. The boxes show the median, upper and lower quartiles of the data, the whiskers extend to the most extreme data point which is no more than 1.5 times the interquartile range from the box. (D) Hazard ratios and 95 % confidence intervals of Cox regression analyses for disease-free survival in metastatic breast cancer patients that received endocrine therapy as next treatment. *$SET_{ER/PR}$* is significantly associated with the survival endpoint over a wide range of different cut-points ranging from the 1st to the 9th decile.

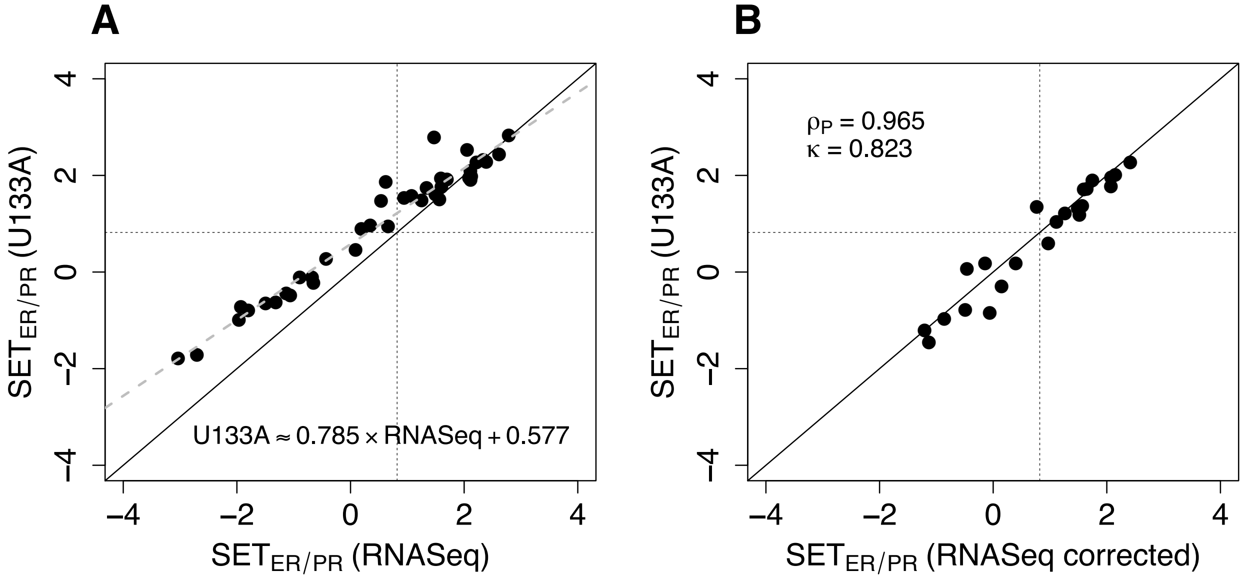

Supplementary Figure 5 Transfer of $SET_{ER/PR}$ to a customized RNA-seq. based assay. In a Calibration study, values from U133A microarray measurements were plotted against the RNA-seq. results (A). Measurements were performed in duplicate on both platforms and a linear model was fit to evaluate platform effects. An independent cohort was used for validation of the estimates (B).
